# Supplementary material for: Identification and functional analysis of non-coding regulatory small RNA FenSr3 in Bacillus amyloliquefaciens LPB-18
Source: PeerJ. 2023 May 15;11:e15236. doi: 10.7717/peerj.15236 (PMC10194069; doi:10.7717/peerj.15236)
Supplement: Supplemental Information 4 [file peerj-11-15236-s004.zip › KO/CK-vs-T1_map/map04380.html]

KEGG PATHWAY: Osteoclast differentiation - Reference pathway


|  |  |
| --- | --- |
| **Osteoclast differentiation - Reference pathway** |  |

[
Pathway menu
| Organism menu
| Pathway entry
| Show description
| User data mapping
]

The osteoclasts, multinucleared cells originating from the hematopoietic monocyte-macrophage lineage, are responsible for bone resorption. Osteoclastogenesis is mainly regulated by signaling pathways activated by RANK and immune receptors, whose ligands are expressed on the surface of osteoblasts. Signaling from RANK changes gene expression patterns through transcription factors like NFATc1 and characterizes the active osteoclast.

|  |  |  |
| --- | --- | --- |
| Reference pathway | 184% 150% 122% 100% 82% 67% 55% | 图片下载 |
